# Mesenchymal Stem Cells from Rats with Chronic Kidney Disease Exhibit Premature Senescence and Loss of Regenerative Potential

**DOI:** 10.1371/journal.pone.0092115

**Published:** 2014-03-25

**Authors:** Barbara Mara Klinkhammer, Rafael Kramann, Monika Mallau, Anna Makowska, Claudia Renate van Roeyen, Song Rong, Eva Bettina Buecher, Peter Boor, Katarina Kovacova, Stephanie Zok, Bernd Denecke, Esther Stuettgen, Simon Otten, Juergen Floege, Uta Kunter

**Affiliations:** 1 Division of Nephrology and Immunology, RWTH Aachen University Hospital, Aachen, Germany; 2 Institute of Pathology, RWTH Aachen University Hospital, Aachen, Germany; 3 Institute of Molecular Biomedicine, Comenius University, Bratislava, Slovakia; 4 Interdisciplinary Centre for Clinical Research, RWTH Aachen University Hospital, Aachen, Germany; National Cancer Institute, United States of America

## Abstract

Mesenchymal stem cell (MSC) transplantation has the potential for organ repair. Nevertheless, some factors might lessen the regenerative potential of MSCs, e.g. donor age or systemic disease. It is thus important to carefully assess the patient's suitability for autologous MSC transplantation. Here we investigated the effects of chronic kidney disease (CKD) on MSC function. We isolated bone marrow MSCs from remnant kidney rats (RK) with CKD (CKD-RK-MSC) and found signs of premature senescence: spontaneous adipogenesis, reduced proliferation capacity, active senescence-associated-β-galactosidase, accumulation of actin and a modulated secretion profile. The functionality of CKD-RK-MSCs *in vivo* was tested in rats with acute anti-Thy1.1-nephritis, where healthy MSCs have been shown to be beneficial. Rats received healthy MSCs, CKD-RK-MSC or medium by injection into the left renal artery. Kidneys receiving healthy MSCs exhibited accelerated healing of glomerular lesions, whereas CKD-RK-MSC or medium exerted no benefit. The negative influence of advanced CKD/uremia on MSCs was confirmed in a second model of CKD, adenine nephropathy (AD). MSCs from rats with adenine nephropathy (CKD-AD-MSC) also exhibited cellular modifications and functional deficits *in vivo*. We conclude that CKD leads to a sustained loss of *in vitro* and *in vivo* functionality in MSCs, possibly due to premature cellular senescence. Considering autologous MSC therapy in human renal disease, studies identifying uremia-associated mechanisms that account for altered MSC function are urgently needed.

## Introduction

As many, mostly positive, results of studies employing mesenchymal stem cell (MSC) therapy for treatment of experimental acute kidney injury (AKI) [1], [2], [3] have been reported, this therapeutic approach has entered clinical evaluation (see www.clinicaltrials.gov NCT00733876, NCT01275612). However, chronic kidney disease (CKD) is a growing public health issue affecting up to 10% of the general population, and once chronic renal replacement therapy becomes necessary, it also represents a massive socioeconomic burden. Nevertheless, the greatly anticipated step to extend clinical MSC studies to progressive CKD is still pending.

Non-malignant MSC maldifferentiation (adipogenic or osteogenic [4], [5]) and the adverse profibrotic side effects [6] have raised concerns about MSC therapy in the setting of CKD. CKD is also relevant in the setting of AKI, as CKD is the most important risk factor for AKI. So far, however, outcomes of preclinical studies on stem and progenitor cell therapy in CKD are inconsistent [7], [8], [9], [10]. In CKD, precise timing of therapy initiation and long-term extension of the therapeutic intervention may be required. In addition, injected, healthy donor-derived cells are suddenly exposed to an altered milieu of various stages of uremia. Besides the accumulated uremic toxins, vitamin D and erythropoietin deficiency, hypertension and acidosis may influence naïve MSCs in their new environment and cause damage that overrides their repair mechanisms.

At present, little is known about the effects of CKD on MSC function. In the present study, we have therefore investigated the potential effects of progressive CKD on MSC functionality.

## Methods

### Harvest, culture and labeling of MSCs

Inbred male F344 rats weighing 180 to 210 g (Harlan Winkelmann, Horst, the Netherlands; or Charles River, Erkrath, Germany) were used as donors for healthy MSCs (H-MSC). In selected experiments, healthy male R26-hPLAP rats (F344 background) that ubiquitously express human placenta alkaline phosphatase (hPLAP), served as transgenic cell donors (TG-MSC) to allow lineage tracking [11]. hPLAP was detected using a rabbit polyclonal IgG antibody (1∶50; AbD Serotec, Oxford, UK) and enzymatically by 5-bromo-4-choloro-3-indolyl phosphate (BCIP) staining in 40% ethanol (EtOH)-fixed tissue as described [11]. For alternative *in vivo* tracking, MSCs were labeled using the PKH26 red fluorescent cell linker kit (Sigma-Aldrich, Saint Louis, MO) as published [12].

MSCs (from healthy donors “H-MSC”, healthy hPLAP-transgenic donors “TG-MSC”, healthy old donors “old donor MSCs”, remnant kidney rats with moderate CKD “CKDmod-RK-MSC”, remnant kidney rats with severe CKD “CKDsev-RK-MSC”, from rats with adenine nephropathy “CKDsev-AD-MSC”) were isolated and cultured as described [5], [12]. Expression of immunophenotypic markers was verified by immunocytochemistry on MSC cytospins. The following primary antibodies were used: mouse monoclonal antibodies against rat CD31 (clone TLD-3A12; 1∶100; AbD Serotec, Oxford, UK), rat CD34 (clone ICO115; 1∶100; Santa Cruz Technology, Heidelberg, Germany), CD44 (clone OX-50; 1∶500; AbD Serotec, Oxford, UK) rat CD73 (clone 5F/B9; 1∶200; BD Biosciences, Heidelberg, Germany) and rat CD90 (clone OX-7; 1∶100; Mediagnost, Reutlingen, Germany) as well as a polyclonal goat-anti-mouse CD45 antibody (clone M-20; 1∶200; Santa Cruz). Osteogenic and adipogenic differentiation of MSCs was tested as described previously [5].

Conditioned media (CM) were obtained by collecting supernatants from confluent MSCs in Passage 2 (P2) (ca. 0.85*10^5^ cells/ml CM) after 48-h culture without fetal calf serum (FCS). VEGF concentrations in the CM were measured (839±90 pg/ml VEGF, n = 3) to ensure accumulation of MSC-secreted factors. Controls consisted of normal FCS free-growth medium [12], [13].

### Determination of MSC proliferation and actin expression

MSC population doublings were calculated as described [14]. In brief, cells were seeded in 12-well plates and the initial seeding number was determined. Triplicates were then trypsinized and counted (multiparameter cell counter CASY TT, Schärfe Systems, Reutlingen, Germany) after 24, 48, 72 and 96 h.

To determine cellular actin content, total cellular protein lysates were prepared and quantified as described. Four microgram of protein was subjected to sodium dodecyl sulfate-polyacrylamide gel electrophoresis (SDS-PAGE), and western blotting was performed as described previously [15] (for the detailed method see Supplementary File S1) using the following antibodies: anti-actin mouse monoclonal antibody (1∶1000; sc-8432, Santa Cruz Biotechnology, Santa Cruz, USA) and horseradish peroxidase-conjugated horse-anti-mouse antibody (Vector Laboratories, Burlingame, CA, USA). Blots were reprobed with a mouse monoclonal antibody for glyceraldehyde-3-phosphate dehydrogenase (GAPDH) (1∶1000; sc-32233, Santa Cruz Biotechnology, Santa Cruz, USA). Stripping was necessary as the proteins of interest (actin and GAPDH) have a predicted size of 42 and 36–40 kD, respectively. Band intensities were quantified by *Scion* Image software (Scion Corporation, USA), and after normalization against values determined for glyceraldehyde 3-phosphate dehydrogenase (GAPDH), the actin content in healthy control H-MSC was set as 1 and relative band intensities were calculated.

### Senescence-associated-β-galactosidase (SA-β-gal) staining and SA-β-gal activity assay

MSCs in P2 and P3 were seeded onto chamber slides (LAB-TEK, Nalge Nunc International, Naperville, IL) at a density of 5*10^3^ cells/cm^2^ and cultured until 80–90% confluency. Cells were washed in PBS and fixed in 0.5% glutaraldehyde for 10 min. MSCs were incubated for 4 h at 37°C with fresh staining solution containing 0.1% x-Gal-solution (5-bromo-4-chloro-3-indolyl β-galactosidase; Sigma, Germany), 40 mM citric acid/sodium-phosphate pH 6, 5 mM potassium ferrocyanide, 5 mM potassium ferricyanide, 150 mM NaCl and 2 mM MgCl_2_. The cells were then counterstained with eosin and mounted with Roti-Histokitt (Carl Roth, Karlsruhe, Germany).

Quantification of SA-β-gal activity was performed using a commercial fluorometric cellular senescence assay kit (Cell Biolabs inc., San Diego, CA, USA). MSCs from healthy and CKDsev-RK donors in Passage 3 were harvested using the lysis buffer provided in the kit, and the assay was performed according to the manufacturer's instructions.

### Reverse transcription-quantitative PCR (RT-qPCR)

Total RNA isolation from MSCs and cDNA synthesis was performed as described [16]. Reverse transcription-quantitative polymerase chain reaction PCR (RT-qPCR) was carried out using an ABI Prism 7300 sequence detector (Applied Biosystems, Weiterstadt, Germany). In each reaction, 0.75 µl of cDNA was amplified in a 25-µl volume using the qPCR Core Kit for SYBR Green I (Eurogentec, Seraing, Belgium). The PCR conditions were 50°C for 2 min followed by 40 cycles of 95°C for 15 sec and 60°C for 1 min. Taqman primers were designed from sequences in the GenBank database using the *Primer Express* software (Applied Biosystems). Primer sequences are listed in Supplementary Table S1. GAPDH cDNA amplification was used as an internal standard.

### Analyses of cytokines, growth factors and carbonyl proteins in MSC culture supernatants

Supernatants of confluent MSCs were used to analyse VEGF_164_ and TGF-β1 by ELISA (Quantikine ELISA, R&D Systems, Minneapolis, MN, USA). Fresh Dulbecco's modified media (DMEM) medium with all additives including FCS was used as control. All experiments were performed in triplicate.

Relative levels of multiple cytokines in culture supernatants of confluent MSCs in Passages 2 and 3 were measured using a commercial kit (Proteome Profiler Antibody Arrays, Rat Cytokine Array Panel A Array Kit, R&D Systems, Wiesbaden-Nordenstadt, Germany). X-ray-films were analyzed using *ImageJ* 1.45 software (Wayne Rasband, NIH, USA). Means for every cytokine were calculated from the duplicate spots from each sample and normalized to the positive controls provided.

Carbonyl proteins were assessed in MSC culture supernatants and lysates by carbonyl protein ELISA (Immundiagnostik AG, Bensheim, Germany).

### MSC-conditioned medium fibroblast stimulation assay

To assess the effects of MSCs on matrix proteins in fibroblasts, the rat fibroblast cell line NRK-49F [17] was stimulated with conditioned medium from H- and CKD-RK-MSC. The conditioned medium was harvested from confluent MSCs (Passage 2) after 3 days of incubation. NRK-49F were seeded into 6-well plates at a density of 40%. Then, 24 h after plating, the culture medium (DMEM+5% FCS) was replaced with starving medium (RPMI+1% P/S) and cells were cultured for another 72 h. Subsequently, NRK were stimulated for 24 h with conditioned medium from either confluent H-MSC, CKDmod-RK-MSC or CKDsev-RK-MSC (n = 3, each). Collagen type I and III mRNA expression were then assessed by RT-qPCR.

### Animal models

Animals were housed under standard conditions (SPF-free) in a light-, temperature- and humidity-controlled environment with free access to tap water and standard rat chow. All animal protocols were approved by the local government authorities [*Landesamt für Natur, Umwelt und Verbraucherschutz Nordrhein Westfalen* (LANUV NRW) 8.87-50.10.35.08.180 and 87-51.04.2010.A380]. All surgeries were performed under ketamin/rompun anesthesia, and every effort was made to minimize suffering. Animals were sacrificed by overdose of isoflurane. A total number of n = 150 animals was used for all experiments (44× remnant kidney model, 8× adenine nephropathy, 24× healthy donors, 6× healthy old donors, 68× anti-Thy1.1-nephritis).

### Rat remnant kidney model

In each of the male F344 rats, weighing 150–180 g (Charles River, Erkrath, Germany), 5/6 nephrectomy (remnant kidney model) was performed under ketamin/rompun anesthesia by right-sided uninephrectomy followed by ligation of two out of three renal arterial branches of the left kidney. This massively reduces the functioning nephrons and induces hypertrophy in the remaining nephrons leading to hypertension, chronic glomerulosclerosis and tubulointerstitial fibrosis culminating in end-stage uremia. Renal function was tested every other week (s-creatinine and s-urea, using an autoanalyzer) and 24-h urine was collected (for proteinuria) from animals housed in metabolic cages. Systolic blood pressure was measured by non-invasive volume pressure recording using the computerized *CODA* system (Kent Scientific Corporation, Torrington, CT) in conscious, trained animals.

### Rat model of adenine nephropathy

Adenine nephropathy was induced in inbred male F344 rats weighing 200–220 g (Charles River, Erkrath, Germany) by 4-week administration of an adenine-rich diet (supplemented with 0.75% adenine) (Altromin, Lage, Germany). This leads to formation of renal crystals within tubuli and tubulointerstitium inducing tubular injury and inflammation, obstruction, necrosis and fibrosis [18]. Renal function was tested every week (s-creatinine and s-urea, using an autoanalyzer) and 24-h urine was collected (for proteinuria) from animals housed in metabolic cages.

### Rat model of acute anti-Thy1.1 nephritis

Acute anti-Thy1.1 nephritis, an experimental model of human IgA nephropathy/mesangioproliferative nephritis (the most frequent immunological renal disease worldwide), was induced in inbred male F344 rats weighing 180–210 g (Charles River, Erkrath, Germany) as described [19]. On Day 2 after disease induction, 0.25*10^6^ MSCs were injected into the left renal artery. Rats received MSCs from healthy wildtype donors (H-MSC), healthy hPLAP-transgenic rats (TG-MSC), rats with remnant kidney (CKDmod-RK-MSC) and rats with adenine nephropathy (CKDsev-AD-MSC). Animals were sacrificed at Day 4 and 6 after disease induction. On Day 5, systolic blood pressure was measured followed by a 24-h urine collection. BrdU (100 mg/kg BW) was injected i.p. 4 h before sacrifice.

In the present studies, the above numbers of injected MSCs are numerically lower than those used in similar experiments in 2006 and 2007 [5], [12]. In retrospect, this relates to a systematic counting error in 2006/2007, and the absolute MSC numbers injected in the present studies are indeed similar.

### Renal morphology and immunohistochemistry

Renal tissue was fixed in methyl Carnoy's solution and embedded in paraffin for light microscopy. Paraffin sections (1 µm) were stained with periodic acid-Schiff reagent and counterstained with hematoxylin. In PAS-stained sections, mesangiolysis scores and the number of total mitotic figures within 100–150 glomerular cross sections were determined [5], [12], [13], [19].

Immunohistochemistry was performed as described [20]. Primary antibodies included a murine monoclonal antibody to α-smooth muscle actin (1∶500; clone 1A4, DAKO Corp., Carpinteria, CA, USA); a murine monoclonal IgG antibody to a cytoplasmic antigen present in monocytes, macrophages and dendritic cells (1∶500; clone ED-1, Serotec, Oxford, UK); a murine monoclonal anti-BrdU antibody (1∶50; clone BU-1, GE Healthcare, Freiburg, Germany) and a goat polyclonal antibody to human collagen type I (1∶100; Southern Biotech, Birmingham, AL, USA). Morphological changes were quantified by computer-assisted image analysis as described [21]. All tissue evaluations were performed in a blinded manner by a single investigator. For histological evaluation of the kidneys of rats with Thy1.1 nephritis, the mean of the right kidneys in the medium group was set as 100%. The right kidneys in all other treatment groups (Healthy, TG, CKD, Adenine) were then normalized to the right kidneys from the control group.

### Statistical analyses

All values are presented as means ± standard deviation. Statistical significance was evaluated using Student's t-test (when comparing two groups) or one-way analysis of variance (ANOVA) with the modified t-test performed with Tukey correction (when comparing more than two groups). Paired t-tests were used to compare the left and right kidneys of an animal.

## Results

### MSC phenotype and morphology

MSCs from normal F344 rats as well as R26-hPLAP rats exhibited characteristic MSC features including spindle-shaped morphology. All MSCs fulfilled the minimal criteria for mesenchymal stromal cells, that is, plastic adherence, inducible osteo- and adipogenesis and specific surface expression patterns. In R26-hPLAP MSCs (transgenic MSCs; TG-MSC), the transgenic enzyme was detectable in the BCIP reaction *in vitro* and *in vivo* as well as by immunohistochemistry for hPLAP (Supplementary Figure S1).

### Donor rats with remnant kidney or adenine nephropathy show moderate to severe CKD

**Figure 1:**
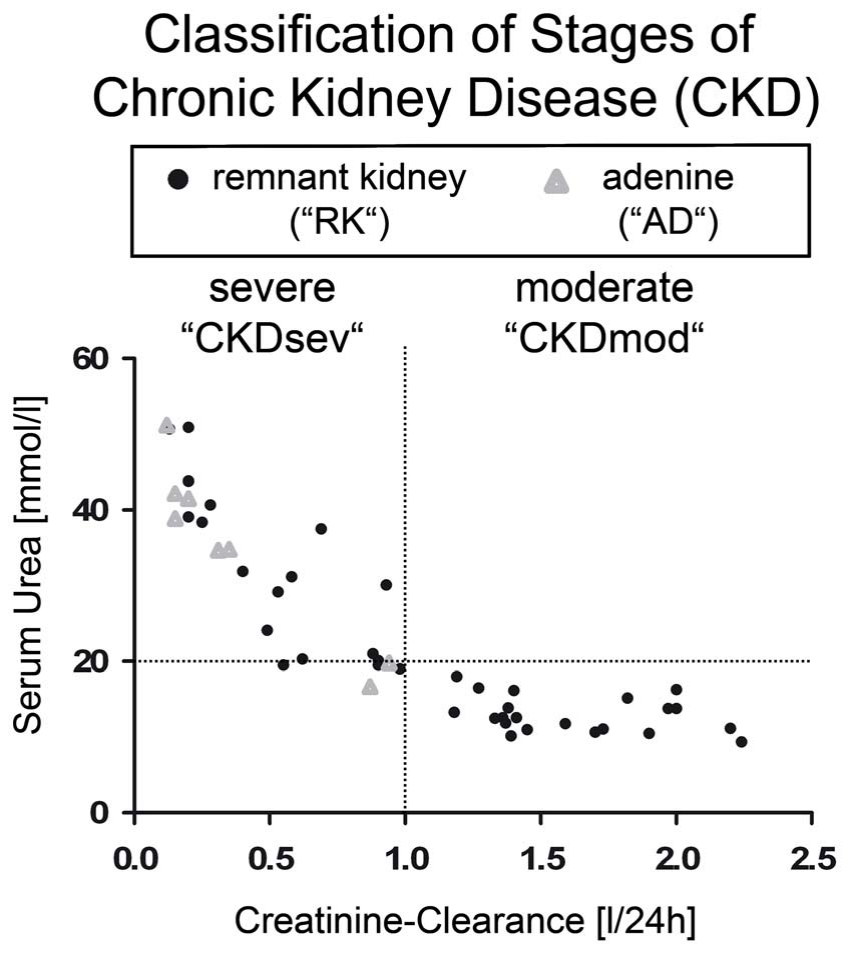
Classification of stages of CKD in rats. All remnant kidney rats (“RK”) were sacrificed after a renal disease duration >17 weeks (mean life expectancy of a healthy lab rat ≈75 weeks). All animals had elevated serum urea and serum creatinine levels at the time of sacrifice. We chose s-urea as a marker for overall uremia and calculated creatinine-clearance to divide the animals into two groups: rats with serum urea >20 mmol/l+creatinine-clearance <1.0 l/24 h (CKDsev-RK) and rats with serum urea ≤20 mmol/l and creatinine-clearance >1.0 l/24 h (CKDmod-RK). Rats with adenine nephropathy (4-week diet containing 0.75% adenine, “AD”) also showed a markedly decreased renal function (CKDsev-AD).

MSCs were isolated from the remnant kidney and adenine nephropathy rats with various degrees of CKD, as evidenced by elevated s-urea and/or a reduced creatinine-clearance (Figure 1, representative histologies are shown in Supplementary Figure S2). We arbitrarily divided the MSCs into two groups: “CKDmod” for moderate renal insufficiency and “CKDsev” for more severe uremia.

### MSCs from remnant kidney rats exhibit mildly altered secretomes compared to healthy MSCs in vitro

MSCs were isolated from remnant kidney rats (CKD-RK-MSC). Secretome of CKD-RK-MSC was compared to healthy donor MSCs (H-MSC) and healthy hPLAP-transgenic-MSCs (TG-MSC) and assessed *in vitro*.

Secretion profiles of H-MSC, TG-MSC and MSCs of remnant kidney donors with CKDmod or CKDsev were analysed in a cytokine array. All MSCs produced high amounts of VEGF_164_ and low levels of fractalkine, CXCL7 and TNFα. While H-MSC tended to produce the highest concentrations of all tested cytokines, a statistically significant difference was only noted for MIP-1α (H-MSC 77±58 vs. CKDsev-RK-MSC 0±18; p = 0.026) (Supplementary Figure S3).

**Figure 2:**
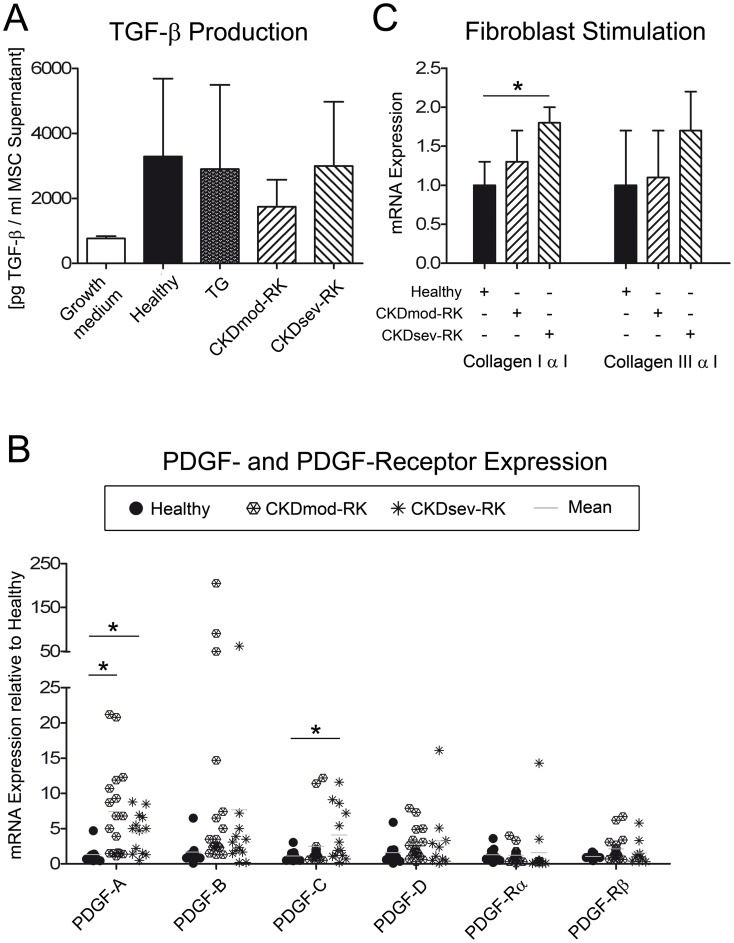
Secretory phenotype of MSCs from healthy and CKD donors. (A) ELISA for activated TGF-β in conditioned medium from MSC in Passage 2 or 3. Supernatants of CKDmod-RK-MSC (n = 8) contained less TGF-β compared to healthy wildtype (healthy, n = 6) or healthy transgenic (TG, n = 4) MSC. Culture medium was used as control (n = 2) (CKDsev-RK-MSC, n = 3). (B) PDGF- and PDGF-receptor expression in H-MSC (n = 9), CKDmod-RK-MSC (n = 19) and CKDsev-RK-MSC (n = 11): PDGF-A and PDGF-C expression is significantly higher in CKDsev-RK-MSC compared to H-MSC. CKDmod-RK-MSC also express significantly more PDGF-A than H-MSC. (C) RT-qPCR for collagen types I and III in NRK49-F fibroblasts stimulated with conditioned medium from healthy MSC (H-MSC) or CKD-MSC for 24 h (n = 3, each). Supernatants from CKDsev-RK-MSC induced a significant increase of collagen type I production in NRK cells. * p<0.05. All data: mean ± SD.

Next we investigated growth factors with a well-established function in glomerular disease. Levels of activated TGF-β in supernatants from CKD-RK-MSC were non-significantly reduced compared to H- or TG-MSC (Figure 2A). Expression of mRNA for PDGF-A and PDGF-C in CKDsev-RK-MSC was significantly higher than in H-MSC. The CKDmod-RK-MSC expressed more PDGF-A compared to H-MSC and tended to produce higher levels of PDGF-B (Figure 2B). Quantification of VEGF_164_ did not show any differences (ELISA data, not shown).

Whether the above differences in secretory activity of CKD-RK-MSC might result in a pro-fibrotic phenotype of MSCs was investigated in NRK cells *in vitro*. Compared to H-MSC, cell culture supernatants from CKDsev-RK-MSC induced significantly higher collagen type I expression in NRK cells after 24 h of stimulation, whereas the expression of collagen type III was not altered (Figure 2C).

### MSCs from remnant kidney rats exhibit cellular senescence

**Figure 3:**
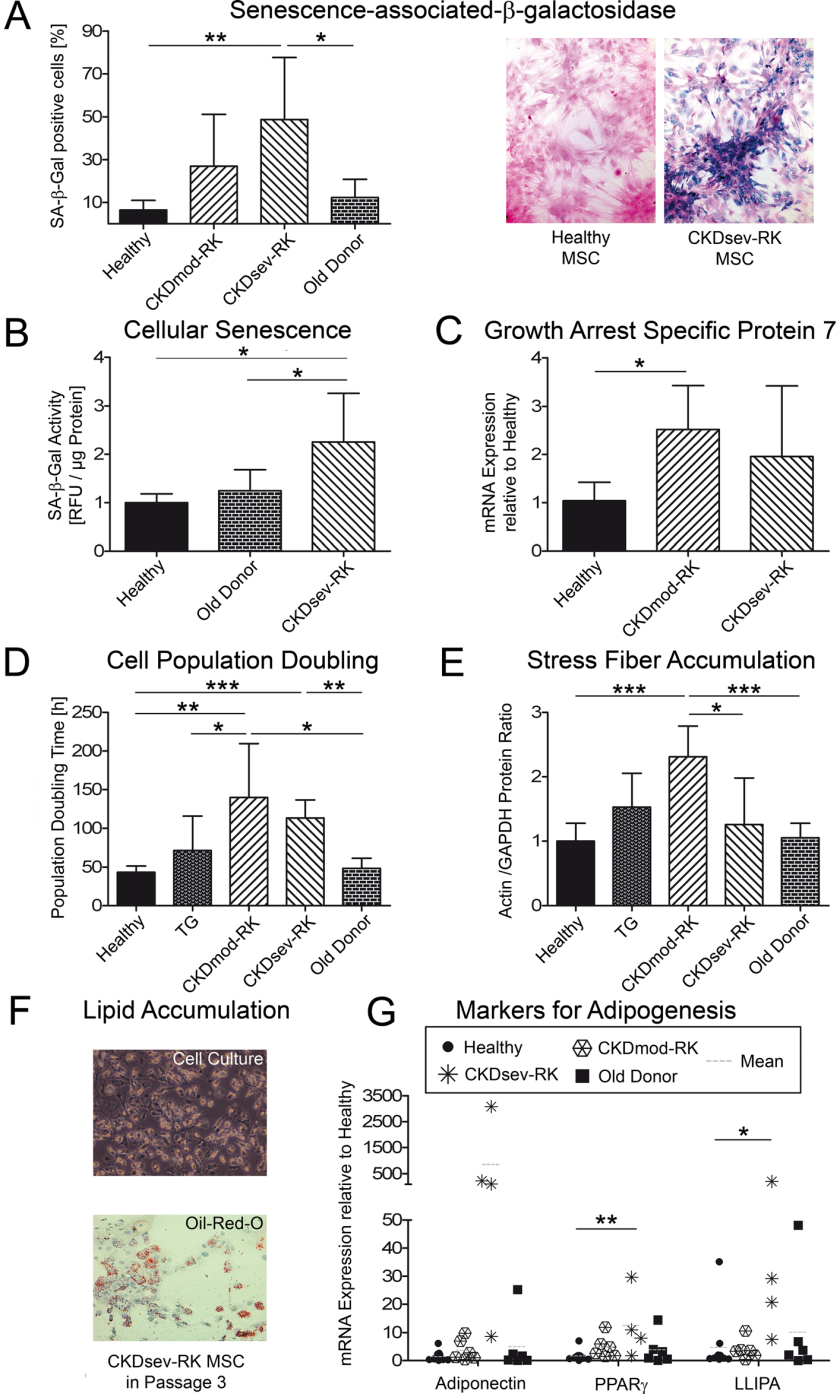
Premature senescence in MSCs from remnant kidney rats. (A) Quantification of enzymatic staining for SA-β-gal in H-MSC (n = 7), CKDmod-RK-MSC (n = 7), CKDsev-RK-MSC (n = 8) and MSCs from healthy old donors (n = 4). Significantly more CKDsev-RK-MSC contain active SA-β-gal compared to H-MSC or MSCs from old donors (p = 0.002 and p = 0.036, respectively). Representative pictures of SA-β-gal staining in H-MSC and CKDsev-RK-MSC are shown (magnification 200×). (B) SA-β-gal activity is also significantly higher in CKDsev-RK-MSC (n = 5) than in H-MSC (n = 6) or MSCs from old donors (n = 6). (C) Expression of Gas7 mRNA (growth-arrest-specific protein 7) in H-MSC (n = 5), CKDmod-RK-MSC (n = 5) and CKDsev-RK-MSC (n = 5). CKDmod-RK-MSC produce significantly more Gas7 mRNA compared to H-MSC (p = 0.01). (D) Cell population doubling time (Passage 2) is significantly higher in all CKD-MSCs (CKDmod-RK (n = 15), CKDsev (n = 4)) than in H-MSC (n = 6), TG-MSC (n = 7) or MSCs from old donors (n = 4). (E) Western blots demonstrate that CKDmod-RK-MSC contain significantly more actin than H-MSC or MSCs from old donors (H-MSC n = 7, TG-MSC n = 4, CKDmod-RK-MSC n = 6, CKDsev-RK-MSC n = 6, healthy old controls (>9 months) n = 5). (F) CKDsev-RK-MSC in Passage 3 spontaneously differentiate into adipocytes (native cell culture image, magnification 200×). Lipid vacuoles are visualized by oil red O staining. Magnification 200×. (G) RT-qPCR for markers of adipogenesis (adiponectin, peroxisome proliferator-activated receptor γ (PPARγ), lipoprotein lipase (LLIPA)) in H-MSC (n = 11), CKDmod-RK-MSC (n = 6), CKDsev-RK-MSC (n = 4) and healthy MSCs from old donors (n = 6). mRNA expression of PPARγ and LLIPA is significantly increased in CKDsev-RK-MSC vs. H-MSC (p = 0.008 and p = 0.03, respectively). All MSCs in Passage 3. * p<0.05; ** p<0.01; *** p<0.001. All data: mean ± SD.

Active senescence-associated-β-galactosidase (SA-β-gal) is a well-known biomarker for senescent cells [22]. In CKDsev-RK-MSC, a significantly higher percentage of cells contained active SA-β-gal and higher SA-β-gal-activity compared to H-MSC or MSCs from age-matched healthy donors (Figure 3A–B). CKDmod-RK-MSC expressed significantly more Gas7 than H-MSCs (growth-arrest-specific protein 7, Figure 3C). MSCs from CKD-RK donors also exhibited a significant increase in cell population doubling time, i.e. loss of proliferating capacity, compared to H-MSC, TG-MSC and MSC from aged healthy donors (old-donor MSC; age >nine months) (Figure 3D).

Actin accumulation, i.e. stress fibers, another marker of senescence, was significantly more frequent in CKDmod-RK-MSC compared to H-MSC and healthy MSCs from old donors (Figure 3E and Supplementary Figure S4). This was no longer the case in CKDsev-RK-MSC, possibly related to their adipogenic differentiation with intracellular lipid storage (Figure 3F+G). CKD-RK-MSC spontaneously differentiated into lipid droplet-containing cells as confirmed by oil red O staining (Figure 3F and Supplementary Figure S5). RT-qPCR detected upregulation of adiponectin, PPARγ and lipoprotein lipase, all markers of adipogenesis (Figure 3G). This was statistically significant in MSCs from CKDsev-RK rats, which showed an up to 3000-fold higher expression of adiponectin mRNA compared to H-MSC in Passage 3. Lipid accumulation and high activity of SA-β-gal often coincided in CKDsev-RK-MSC. A trend towards adipogenesis, but not osteogenesis, was observed in our MSCs from healthy aged donor rats (Supplementary Table S2), confirming data of others [23]. Only mRNA-levels for osteocalcin (but not for osteopontin or the transcription factor cbfα-1 (core-binding factor subunit α1) were significantly increased in CKD-MSC vs. H-MSC (Supplementary Table S2), corroborating previous findings in human MSCs under uremic conditions [24]. Nevertheless, no apparent spontaneous calcification of CKD-MSC was observed in normal growth medium *in vitro*.

Finally, we assessed carbonyl proteins, a marker of oxidative damage to proteins in CKD-RK-MSC, as a possible cause of MSC senescence or lipid accumulation. However, these did not exhibit significant differences between H-MSC and CKDsev-RK-MSC, which revealed spontaneous lipid accumulation in Passage 2 (supernatants from H-MSC 128±92 (n = 3) vs. CKDsev-RK-MSC 177±94 pmol/mg total protein (n = 6), p = 0.49).

### Transplanted MSCs from remnant kidney donor rats do not accelerate healing in anti-Thy1.1-nephritis

To assess the functionality of CKD-RK-MSC *in vivo*, rats with anti-Thy 1.1 nephritis at Day 2 after disease induction received injections into the left renal artery of H-MSC, TG-MSC, CKDmod-RK-MSC or DMEM. We previously reported that H-MSC ameliorate histological damage and acute kidney injury in such rats [12]. PKH26-labeled MSCs were detected exclusively in the treated left kidneys up to 4 days after injection whereas the right internal control kidneys remained negative. There were no significant differences between the MSC treatment groups regarding the number of engrafted glomerular cells (Supplementary Figure S6).

**Figure 4:**
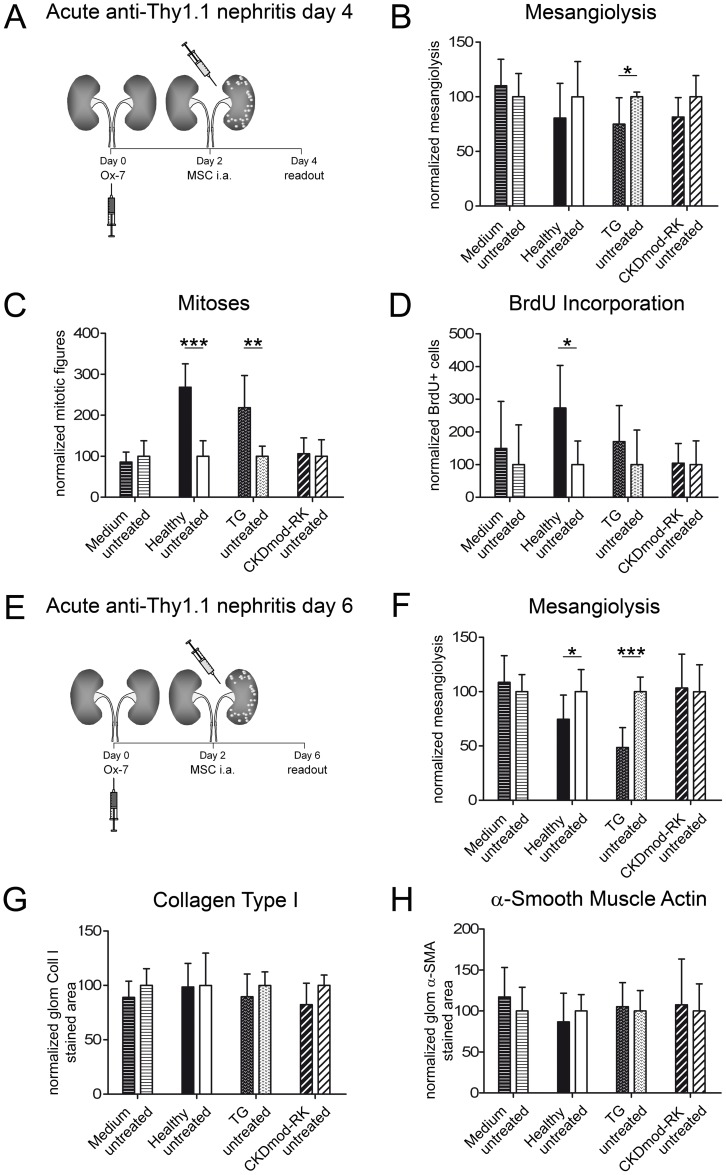
Analysis of renal function and histology on Day 4 and Day 6 of anti-Thy1.1-nephritis. (A) Experimental design. (B–D) Comparison of rats that had anti-Thy1.1-nephritis and received H-MSC (“Healthy”, n = 7), TG-MSC (“TG”, n = 8), CKDmod-RK-MSC (“CKDmod-RK”, n = 6) or control DMEM (“Medium”, n = 10) injected into the left renal artery on Day 2 after disease induction and were analysed on Day 4. (E) Experimental design. (F–H) Comparison of rats that had anti-Thy1.1-nephritis and received H-MSC (“Healthy”, n = 7), TG-MSC (“TG”, n = 7), CKDmod-RK-MSC (“CKDmod-RK”, n = 6) or control DMEM (“Medium”, n = 9) injected into the left renal artery on Day 2 after disease induction and were analysed on Day 6. * p<0.05; ** p<0.01; *** p<0.001. All data: mean ± SD.

On Day 4, i.e. 2 days after MSC-injection (Figure 4A), s-creatinine and s-urea levels were similar in rats receiving H-MSC, TG-MSC or DMEM injections, whereas s-creatinine of CKDmod-RK-MSC-treated rats was significantly elevated (Table 1). Only TG-MSC, but not CKDmod-RK-MSC, significantly reduced mesangiolysis (Figure 4B). Both healthy H-MSC and TG-MSC but not CKDmod-RK-MSC led to a significant increase in glomerular mitotic figures in treated kidneys (Figure 4C), paralleled by increased BrdU uptake (Figure 4D). In all MSC-treated kidneys, the number of ED-1-positive (infiltrating) cells was higher than in the contralateral kidneys, but only in the H-MSC group did this reach statistical significance (Supplementary Figure S7). None of the treatments affected the percentage of the α-SMA-positive glomerular area in the left vs. right kidneys (Supplementary Figure S7).

**Table 1:** Functional parameters of rats with anti-Thy1.1-nephritis on Days 4 and 6 (2 and 4 days after treatment).

Day 4	Medium (n = 10)	H-MSC (n = 7)	TG-MSC (n = 8)	CKDmod-RK-MSC (n = 6)
Serum urea [mmol/L]	7.5±1.7	8.1±1.5	7±0.7	8.8±1.9
Serum creatinine [µmol/L]	52.2±4.4	50±6.6	51.1±5.4	59.3±8.9 *

Day 6	Medium (n = 9)	H-MSC (n = 7)	TG-MSC (n = 7)	CKDmod-RK-MSC (n = 6)	CKDsev-AD-MSC (n = 8)
Syst. blood pressure [mmHg]	128±9	128±15	127±21	130±22	137±10
Serum urea [mmol/L]	6.0±1.0	13.5±5.7 *	11.0±0.9 *	7.9±1.0 *	8.9±1.5 *
Serum creatinine [µmol/L]	39.9±5.9	43.7±5.2	43.6±3.7	41.3±4.7	47±4.9 *
Proteinuria [mg/24 h]	40±25	24±26	9±6 *	34±16	52±21

* p<0.05 compared to Medium group.

Comparison of rats with anti-Thy1.1 nephritis that received healthy MSCs (H-MSC), TG-MSC, CKDmod-RK-MSC, CKDsev-AD-MSC or control DMEM “medium” into the left renal artery on Day 2 after disease induction.

On Day 6, i.e. 4 days after MSC injection (Figure 4E), only few statistical differences in functional parameters persisted between the four treatment groups (Table 1), namely TG-MSC-treated rats showing the lowest proteinuria compared to controls. There were no statistically significant differences in s-creatinine but a trend towards higher s-urea in MSC-treated animals, possibly related to the reduced food intake. Consistent with our prior data [12], both H-MSC and TG-MSC treatment led to a significant reduction of mesangiolysis, mesangiolysis persisted with no trend for healing in rats receiving control medium and CKDmod-RK-MSC (Figure 4F). The three MSC groups failed to exhibit significant differences in the number of glomerular mitoses and influx of monocytes/macrophages (Supplementary Figure S7). No profibrotic action of MSC treatment was noted (α-SMA and collagen-I-positive areas, Figure 4G+H).

### MSCs from rats with adenine nephropathy show alterations similar to MSCs from remnant kidney rats

**Figure 5:**
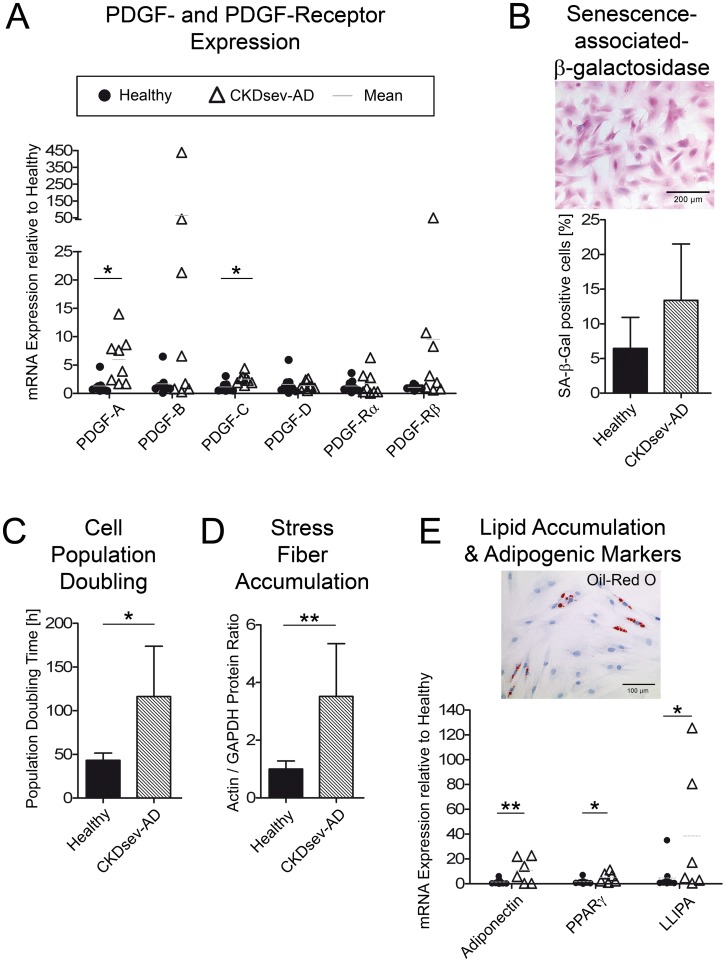
In vitro characterization of MSCs from rats with adenine nephropathy. (A) CKDsev-AD-MSC have a decreased proliferation capacity (cell population doubling time 116.1±57.7 h (n = 6) vs. 43±8.2 h in H-MSC (n = 5); p = 0.02). (B) CKD-sev-AD-MSC (n = 8) express significantly more PDGF-A and PDGF-C than H-MSC (n = 9) (p = 0.008 and p = 0.005 respectively). CKDsev-AD-MSC contained active SA-β-gal (C) and in some cases lipid vacuoles (D). * p<0.05; ** p<0.01; *** p<0.001. All data: mean ± SD.

MSCs were isolated from rats that received a diet supplemented with 0.75% adenine for 4 weeks (s-urea 35±12 mmol/l, creatinine clearance 0.4±0.3 l/24 h, n = 8; “CKDsev-AD-MSC”). Just as CKD-RK-MSC, CKDsev-AD-MSC expressed significantly more PDGF-A and PDGF-C than H-MSC (CKDsev-AD-MSC (n = 8) vs. H-MSC (n = 9): p = 0.008 and p = 0.005, Figure 5A) and contained significantly higher amounts of active SA-β-gal (Figure 5B). CKDsev-AD-MSC showed a significant increase in cell population doubling time compared to H-MSC (116±58 h vs. 43±8 h; p = 0.02; Figure 5C) and contained significantly more actin fibers (Figure 5D). CKDsev-AD-MSC (occasionally) exhibited spontaneous adipogenic differentiation and expressed significantly more mRNA for adipogenic markers (adiponectin, PPARγ and lipoprotein lipase, Figure 5E).

**Figure 6:**
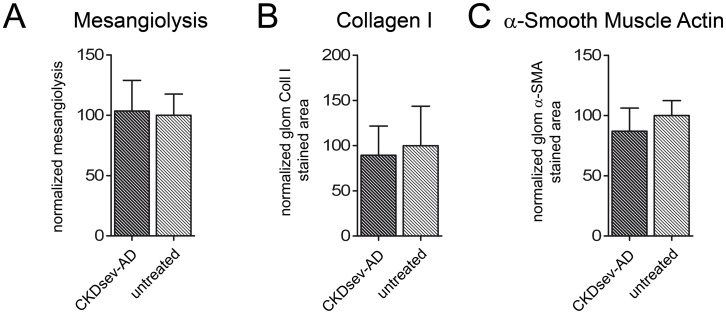
Analysis of renal function and histology on Day 6 of anti-Thy1.1-nephritis, 4 days after treatment with CKDsev-AD-MSC. Left kidneys of rats with acute Thy1.1 nephritis that were treated with CKDsev-AD-MSC did not show significant differences in mesangiolysis (E), glomerular collagen I (F) or α-SMA- positive glomerular area (G) compared to untreated right control kidneys on Day 6. * p<0.05; ** p<0.01; *** p<0.001. All data: mean ± SD.

The *in vivo* functionality of CKDsev-AD-MSC was also analysed in rats with anti-Thy 1.1 nephritis (Figure 6A–C and Supplementary Figure S8). Rats that received CKDsev-AD-MSC had significantly higher s-creatinine than controls (Table 1).

On Day 6, i.e. 4 days after CKDsev-AD-MSC injection, neither mesangiolysis (treated vs. untreated kidney: p = 0.71) nor the influx of ED-1-positive cells (p = 0.18) were reduced in treated kidneys. CKDsev-AD-MSC did not increase glomerular mitoses (p = 0.48) or the α-SMA and collagen-I-positive glomerular areas (p = 0.08 and 0.25, respectively) in the left vs right kidneys.

## Discussion

Studies on cell-based regenerative therapies employing MSCs or other progenitors from healthy donors in animal models of CKD have yielded conflicting results [7], [8], [9], [10], [25], [26], the reasons remaining unclear. In this study, we assessed whether the uremic millieu itself might alter MSC function by causing premature stem cell aging, i.e. senescence. Stem cells are the longest living cells in the mammalian proliferative compartment, implicating an increased risk for the development of mutations or epigenetic changes that could lead to malignant transformation. Senescence has emerged as a mechanism to avoid potentially harmful proliferation of damaged stem cells. We therefore tested the influence of CKD on rat bone marrow-derived MSCs: phenotype, secretome, differentiation capacity and proliferation rates. In addition, to the best of our knowledge, we were the first to isolate CKD-MSCs from a large number of animals, and two different models of CKD, and to use these cells *in vivo* to test for their regenerative potential in acute anti Thy1.1 nephritis.

Our first major finding was that CKD-MSCs obtained from rats with two different models of CKD, namely the remnant kidney model and adenine nephropathy, *in vitro* do indeed exhibit many signs of premature senescence, in particular markedly reduced proliferation rates, stress fiber accumulation and spontaneous adipogenesis *in vitro*. The latter can, retrospectively, explain our (much discussed) observation of intraglomerular adipogenic maldifferentiation after intrarenal MSC injection in a chronic nephritis model [13]. In line with our observations, several abnormalities of non-MSC hematopoietic and endothelial precursor cells in CKD have been reported, including a reduced capacity for *in vitro* proliferation in adherent bone marrow progenitor cells [27], genomic damage to CD34+ hematopoietic progenitor cells [28], premature aging of circulating T cells [29] and functional impairment (reduced number in peripheral blood, reduced proliferation capacity *in vitro*) of endothelial precursor cells [30], [31]. In addition, healthy bone marrow transplants have recently been shown to be more beneficial in CKD rats than bone marrow transplants from CKD donors [32]. Normal aging also affects stem cell function. Thus, transplantation of full bone marrow from young donors alleviated renal aging-associated morphology (e.g. collagen IV deposition, SA-β-gal expression) in recipient mice aged 18 months [33]. Most importantly, in the context of our data, there are also very recent data on an *in vitro* functional impairment of bone marrow stromal cells from mice after 6 weeks of mild CKD [34]. As in our study, these cells exhibited cellular senescence but, in contrast to our data, no reduction in proliferation rates until Passage 11. Nevertheless, these cells were not tested for their renal regenerative potential *in vivo*.

Premature MSC senescence induced by CKD was “dose-dependent” in our study, i.e. MSCs from sicker animals (CKDsev-RK-MSC) exhibited senescence as early as Passage 2. This may be an important explanation for the variable effects observed in MSC-CKD studies. Given that the non-uremic cell culture conditions did not reverse the MSC phenotype after *in vivo* exposure to uremic conditions, it is likely that epigenetic changes, similar to those observed in aging MSCs [35], are induced by CKD. Indeed, this may represent a key mechanism of what has been termed “uremic memory” based on clinical observations [36]. Which “uremic factor(s)” (see EUTox, www.uremic-toxins.org) might cause MSC impairment? Many uremic factors have been identified, and it is widely accepted that chronic renal replacement therapy (dialysis) should eliminate these in the best way possible. Indoxyl sulfate and p-cresol decrease proliferation in mouse MSCs [34]. Other factors contributing to stem and progenitor cell damage include reactive oxygen species [37], [38], proinflammatory molecules [39] and angiotensin II [40]. The setting of (successful) renal transplantation best describes a situation where all body cells undergo a sudden change from chronic uremia (comparable to CKDsev) to healthy or CKDmod, depending on graft function. We feel that our results further demonstrate the urgent need for good quality dialysis and early renal transplantation, if applicable.

MSC senescence induced by CKD in our study also altered their secretory profile. Thus, we found a, albeit non-significant, reduced secretion of osteoblast-stimulating (and profibrotic) TGF-β in CKDmod-MSC from remnant kidney rats, which is in line with the “adipogenic switch” [41]. In addition, we noted a significant upregulation of PDGF-A and -C in MSCs from severely uremic rats (RK and AD). PDGFs are key players in the development of renal fibrosis. Moreover, they stimulate MSCs to proliferate [42] but limit their multipotency through regulation of Oct4 and Nanog expression [43] with their overexpression possibly representing a compensatory mechanism given their reduced proliferation rates. Concerning their effect on fibrosis, human MSCs have been shown to ameliorate obstruction-induced renal fibrosis in rats with unilateral ureter ligation [44], but these rats still had a healthy kidney and did not develop CKD, thus the exogenous MSCs remained unaffected by uremia. Others injected healthy donor MSCs weekly into Alport mice with CKD and found no clinical improvement although there was some reduction in renal fibrosis as well as a reduction in loss of peritubular capillaries [45].

MSC preparations can differ substantially. For example, an up to 38-fold difference in the interleukin-1α expression was noted when comparing adipose tissue with bone marrow-derived rat MSC [46]. Beneficial effects of (activated) MSC preparations in highly inflammatory conditions have been tracked down to the secretion of one specific protein (TNFα-stimulated gene 6 protein, TSG6) [47] via decreasing TLR2/NF-κB signaling. Consistent with the above, our data show considerable standard deviation in cytokine expression in MSC supernatants, confirming results in human MSCs [48]. Also, the source of the MSCs highly influences the characteristics of the cells and might explain why first tests did not detect differences between MSCs derived from adipose tissue of CKD patients and healthy donors [49].

It is conceivable that some of the above discrepancies, i.e. some studies demonstrating a benefit from RK-MSC and others showing no benefit, relate to the different therapeutic schemes and MSC dosages or other variables such as rat strains and experimental conditions. However, our data point to a new explanation, which has been neglected so far, namely the effects of CKD on MSC function:

In addition to our *in vitro* findings, we extended our studies to animal experiments and were the first to test the regenerative potential of CKD-MSCs vs. MSCs from healthy donors *in vivo* using the acute anti Thy1-nephritis model. We previously reported that, in this model, MSCs mediate repair mostly via paracrine phenomena and not by differentiation [2], [12]. Senescent cells still secrete many growth factors [23], [50], thus, growth arrest itself does not necessarily mean loss of regenerative potential. Our second major finding is that MSCs derived from animals with CKD (RK or AD) lost the ability to increase glomerular cell proliferation and to thereby reduce mesangiolysis, unlike cells from healthy normal or transgenic donors. Notably, CKD in one set of MSC donors (RK) was “only” moderate. Interestingly, although CKD-RK-MSC supernatants did stimulate rat kidney fibroblast collagen production *in vitro* much more than H-MSC supernatants, we did not find enhanced collagen accumulation in CKD-RK-MSC-treated anti-Thy 1.1 nephritic kidneys. This is in line with the literature showing both pro- and antifibrotic effects of MSCs under different conditions [51], [52].

We conclude that endogenous bone marrow MSCs are functionally impaired by CKD, already in moderate stages and independent of the origin of CKD. This damage cannot be reversed after cell isolation by culture expansion in normal growth medium. Our data suggest that CKD in rat MSCs leads to complex phenotypic changes that are consistent with premature cellular senescence.

Rapid functional alteration of MSCs by CKD might explain why even repeated injections of healthy progenitor cells did not improve CKD in several published animal studies. These findings raise two important questions for translational medicine: first, does it make sense to treat CKD patients with autologous MSCs? If yes, up to which stage and/or duration of CKD? Second, what happens to MSCs from healthy donors after transplantation into a recipient with CKD? Studies to identify the *in vivo* mechanisms leading to MSC damage in CKD and their time course are urgently needed to identify potential protective approaches.

## Supporting Information

Figure S1 **Cell tracking: detection of transplanted TG-MSC in kidney tissue.**
(DOC)

Figure S2 **Renal histology of MSC donors: healthy, remnant kidney (CKDmod-RK, CKDsev-RK), adenine nephropathy (CKDsev-AD) and the respective recipients (anti-Thy1.1-nephritis).**
(DOC)

Figure S3 **Cytokine-Array of MSC supernatants.**
(DOC)

Figure S4 **Cell morphology of healthy and CKD-MSC.**
(DOC)

Figure S5 **Spontaneous and induced differentiation of MSCs.**
(DOC)

Figure S6 **Engraftment of transplanted MSCs.**
(DOC)

Figure S7 **Analysis of renal histology on day 4 or day 6 of anti-Thy1.1-nephritis.**
(DOC)

Figure S8 **Analysis of renal histology on day 6 of anti-Thy1.1-nephritis treated with CKDsev-AD-MSCs.**
(DOC)

File S1 **Western Blot for intracellular accumulation of actin filaments in MSC (method in detail).**
(DOC)

File S2 **ARRIVE Guidelines.**
(DOC)

Table S1 **Primer for RT-qPCR.**
(DOC)

Table S2 **mRNA expression of osteogenic markers in MSCs.**
(DOC)
