# Supplementary material for: Mesenchymal Stem Cells from Rats with Chronic Kidney Disease Exhibit Premature Senescence and Loss of Regenerative Potential
Source: PLoS One. 2014 Mar 25;9(3):e92115. doi: 10.1371/journal.pone.0092115 (PMC3965415; doi:10.1371/journal.pone.0092115)
Supplement: Figure S1 — Cell tracking: detection of transplanted TG-MSC in kidney tissue. (DOC) [file pone.0092115.s001.doc]

**Supplementary Figure S4:**

**Cell tracking: detection of transplanted TG-MSC in kidney tissue**

(A) Immunohistochemical and (B) enzymatic detection of hPLAP-positive intraglomerular MSC after intraarterial injection. Magnification 200x (A), 400x (B).
