# Supplementary material for: Mesenchymal Stem Cells from Rats with Chronic Kidney Disease Exhibit Premature Senescence and Loss of Regenerative Potential
Source: PLoS One. 2014 Mar 25;9(3):e92115. doi: 10.1371/journal.pone.0092115 (PMC3965415; doi:10.1371/journal.pone.0092115)
Supplement: Figure S2 — Renal histology of MSC donors: healthy, remnant kidney (CKDmod-RK, CKDsev-RK), adenine nephropathy (CKDsev-AD) and the respective recipients (anti-Thy1.1-nephritis). (DOC) [file pone.0092115.s002.doc]

**Supplementary Figure S5:**

**Renal histology of MSC donors: healthy, remnant kidney (CKDmod-RK, CKDsev-RK), adenine nephropathy (CKDsev-AD) and the respective recipients (anti-Thy1.1-nephritis)**


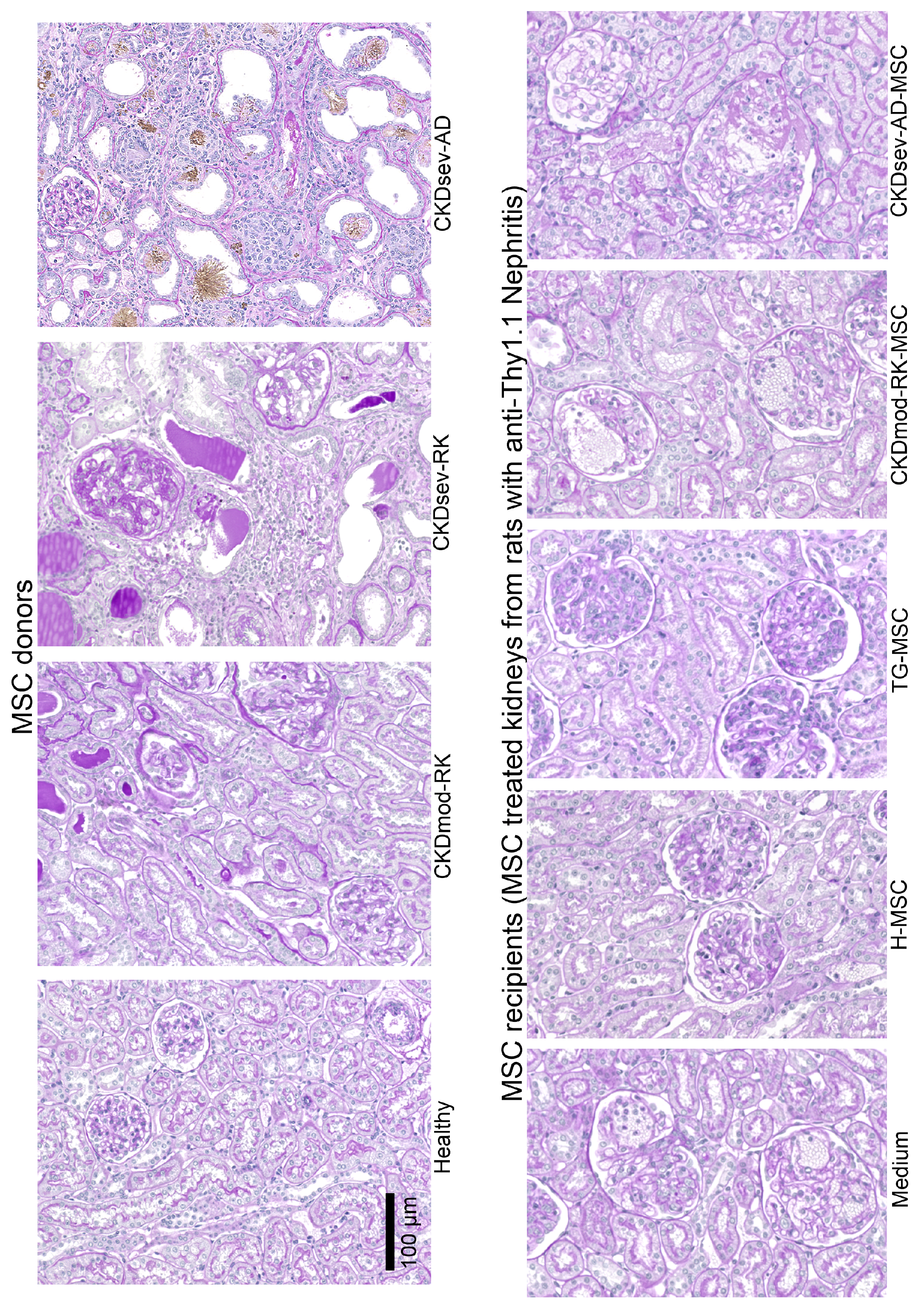


PAS stained sections (1µm) from MSC donor rats and MSC recipients.
