# Supplementary material for: Mesenchymal Stem Cells from Rats with Chronic Kidney Disease Exhibit Premature Senescence and Loss of Regenerative Potential
Source: PLoS One. 2014 Mar 25;9(3):e92115. doi: 10.1371/journal.pone.0092115 (PMC3965415; doi:10.1371/journal.pone.0092115)
Supplement: Figure S3 — Cytokine-Array of MSC supernatants. (DOC) [file pone.0092115.s003.doc]

**Supplementary Figure S6:**

**Cytokine-Array of MSC supernatants**


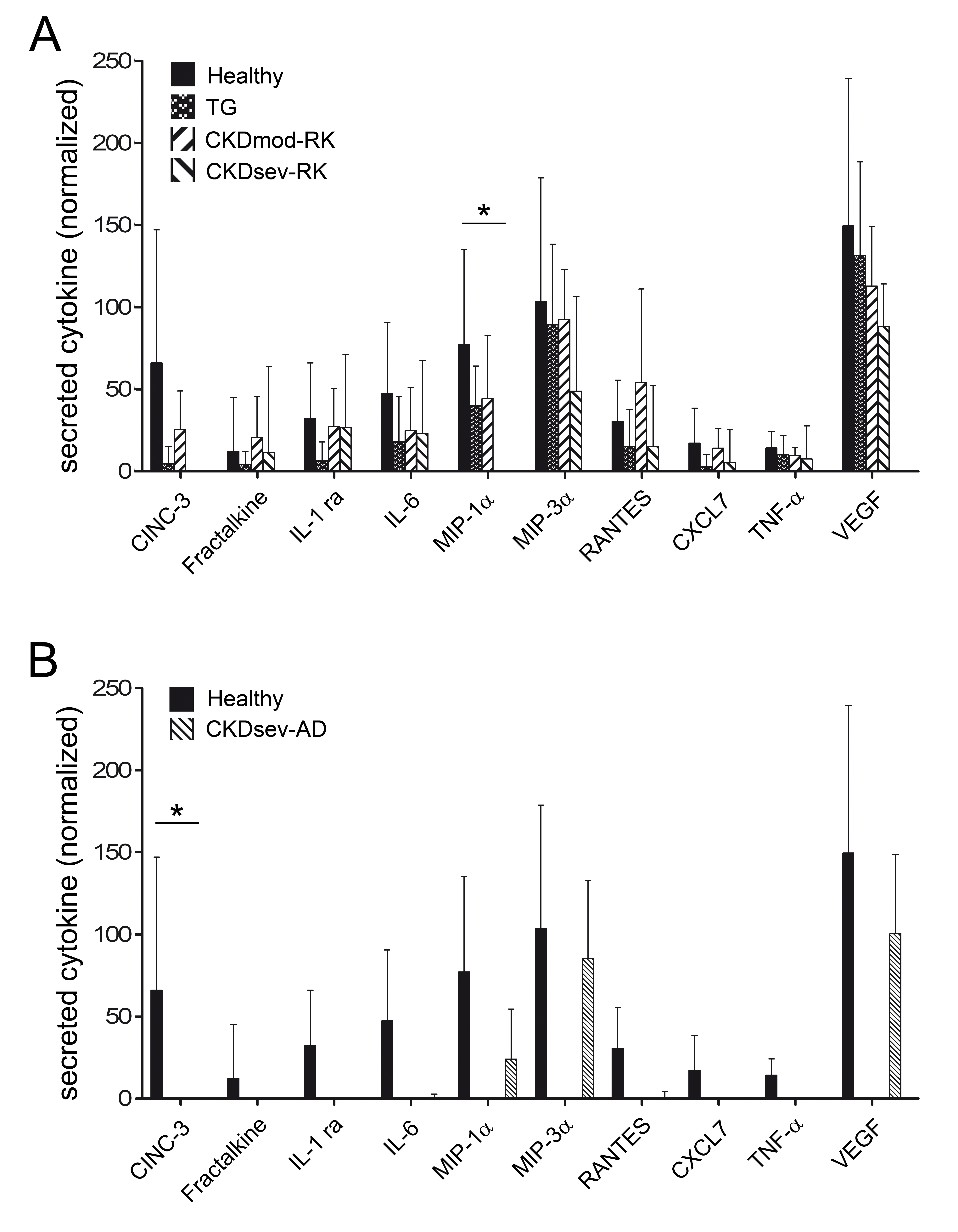


Cytokine array for secretion profiles of different MSCs.

(A) H-MSCs produced significantly more MIP1-α than CKDsev-RK MSCs. (H-MSC (n = 5), TG-MSCs (n = 4), CKDmod-RK-MSCs (n = 8), CKDsev-RK-MSCs (n = 4)). (B) CKDsev-AD-MSCs produced significantly less CINC-3 compared to H-MSCs (H-MSCs (n = 5), CKDsev-AD-MSCs (n = 7)).

Supernatants were used from MSCs in passage 2 or 3.

* p < 0.05. All data: mean ± SD.
