# Supplementary material for: Mesenchymal Stem Cells from Rats with Chronic Kidney Disease Exhibit Premature Senescence and Loss of Regenerative Potential
Source: PLoS One. 2014 Mar 25;9(3):e92115. doi: 10.1371/journal.pone.0092115 (PMC3965415; doi:10.1371/journal.pone.0092115)
Supplement: Figure S4 — Cell morphology of healthy and CKD-MSC. (DOC) [file pone.0092115.s004.doc]

**Supplementary Figure S7:**

**Cell morphology of healthy and CKD-MSC**


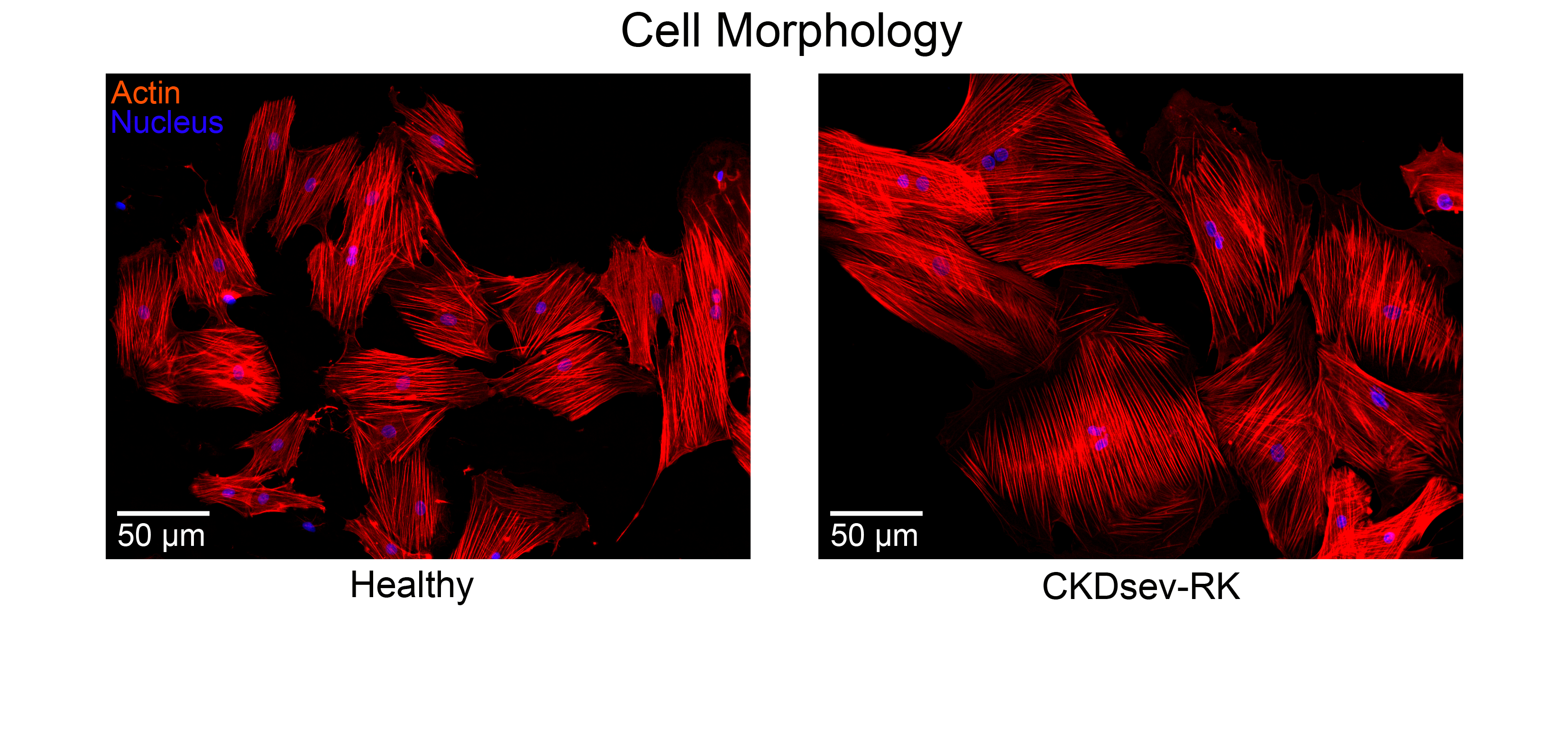


Actin cytoskeleton was stained with Phalloidin-TRITC (red = actin, blue = nuclei).

CKDsev-RK-MSCs are flattened and enlarged compared to healthy H-MSCs.
