# Supplementary material for: Mesenchymal Stem Cells from Rats with Chronic Kidney Disease Exhibit Premature Senescence and Loss of Regenerative Potential
Source: PLoS One. 2014 Mar 25;9(3):e92115. doi: 10.1371/journal.pone.0092115 (PMC3965415; doi:10.1371/journal.pone.0092115)
Supplement: Figure S5 — Spontaneous and induced differentiation of MSCs. (DOC) [file pone.0092115.s005.doc]

**Supplementary Figure S8:**


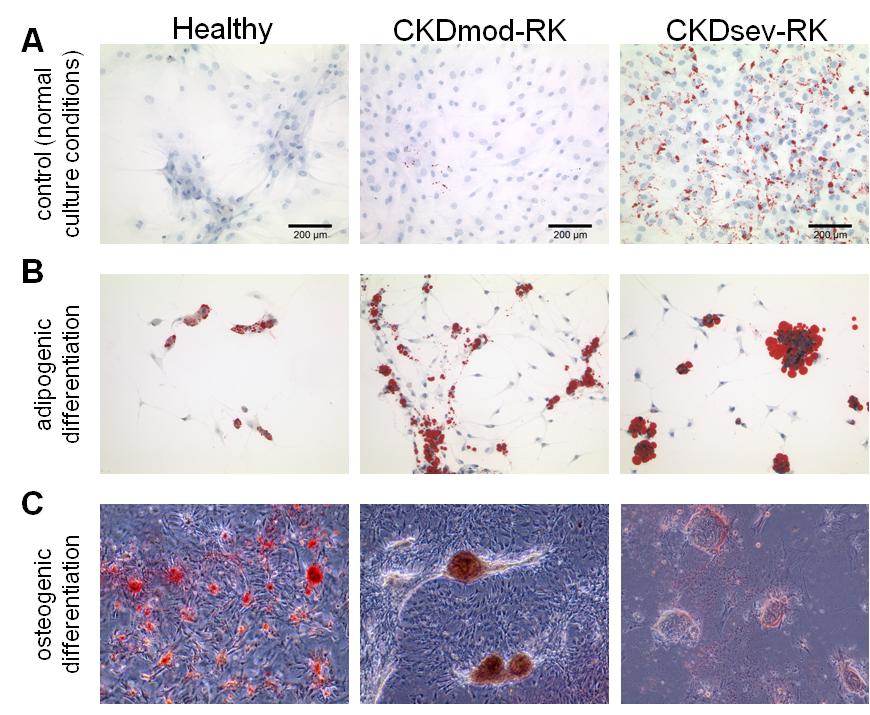
**Spontaneous and induced differentiation of MSCs**

Adipogenic (A+B) and osteogenic (C) differentiation capacity of H-MSCs, CKDmod-RK-MSCs and CKDsev-RK-MSCs.

Oil red O staining confirmed adipogenic differentiation of MSCs (P3) after culture in adipogenic induction medium (B) but also visualized fat vacuoles in CKD-MSCs grown in normal culture medium (A). H-MSCs did not show spontaneous differentiation (A). Healthy as well as CKD-MSCs are also capable of differentiating into and osteoblasts *in vitro* shown by alizarin red staining (C).
