# Supplementary material for: Mesenchymal Stem Cells from Rats with Chronic Kidney Disease Exhibit Premature Senescence and Loss of Regenerative Potential
Source: PLoS One. 2014 Mar 25;9(3):e92115. doi: 10.1371/journal.pone.0092115 (PMC3965415; doi:10.1371/journal.pone.0092115)
Supplement: Figure S6 — Engraftment of transplanted MSCs. (DOC) [file pone.0092115.s006.doc]

**Supplementary Figure S9:**

**Engraftment of transplanted MSCs**


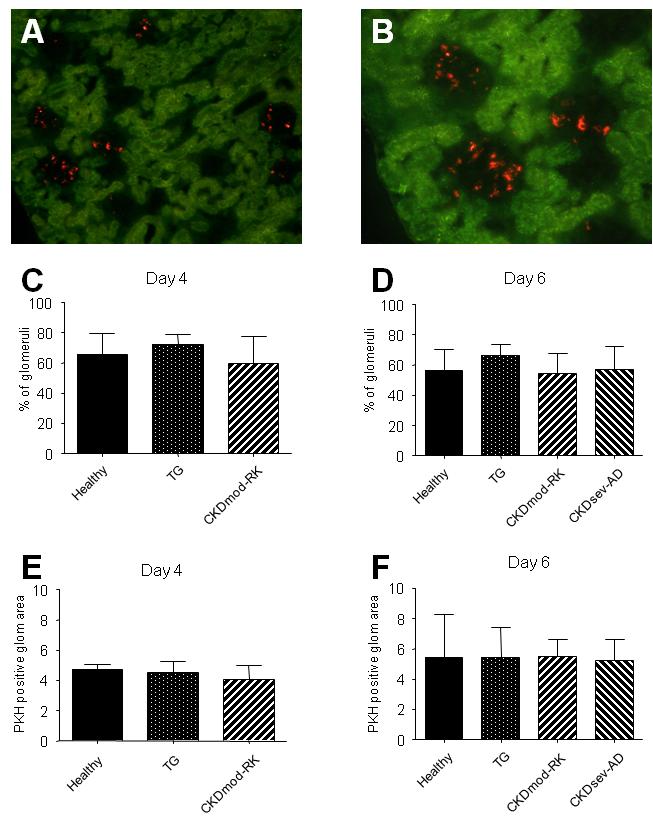


(A-B) Fluorescence microscopy in frozen tissue sections from MSC-treated kidneys for detection of PKH-labeled MSCs. (A+B) Overlay of kidney autofluorescence at 450-490 nm and PKH26 at 515-560 nm (magnification 100x and 200x, respectively). Four days after MSCs injection into the renal artery of rats with anti-Thy1.1-nephritis, bright PKH26 fluorescence is detected in most ipsilateral but not contralateral (data not shown) glomeruli. (C+D) Number of PKH-positive glomerular cross sections and (E+F) quantification of the PKH-positive area in glomeruli in frozen sections from left trated kidneys (day 4: rats that had anti-Thy1.1-nephritis and received H-MSCs ("Healthy", n=7), TG-MSCs ("TG”, n=8), CKD-MSCs (“CKDmod-RK”, n=6); day 6: H-MSCs ("Healthy", n=7), TG-MSCs ("TG”, n=7), CKD-MSCs (“CKDmod-RK”, n=6), Adenine-MSCs (“CKDsev-AD”, n=8)). On day 4 as well as on day 6 there were neither differences in the number of PKH-positive glomeruli (ANOVA day 4: p=0,239; day 6: p=0,376) nor in the area staining positively for PKH per glomerular cross section (ANOVA day 4: p=0,222; day 6: p=0,996). All data: mean ± SD.
