# Supplementary material for: Mesenchymal Stem Cells from Rats with Chronic Kidney Disease Exhibit Premature Senescence and Loss of Regenerative Potential
Source: PLoS One. 2014 Mar 25;9(3):e92115. doi: 10.1371/journal.pone.0092115 (PMC3965415; doi:10.1371/journal.pone.0092115)
Supplement: Figure S7 — Analysis of renal histology on day 4 or day 6 of anti-Thy1.1-nephritis. (DOC) [file pone.0092115.s007.doc]

**Supplementary Figure S10:**

**Analysis of renal histology on day 4 or day 6 of anti-Thy1.1-nephritis**

Day 4 (2 days after treatment):


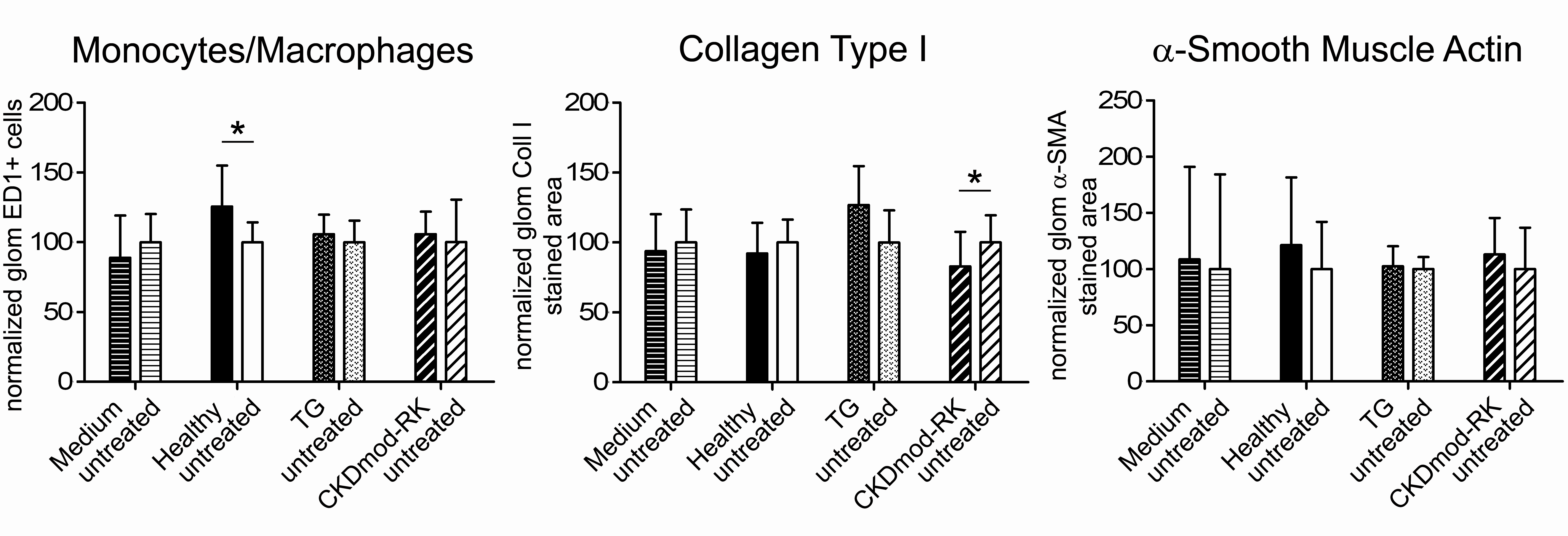


Day 6 (4 days after treatment):


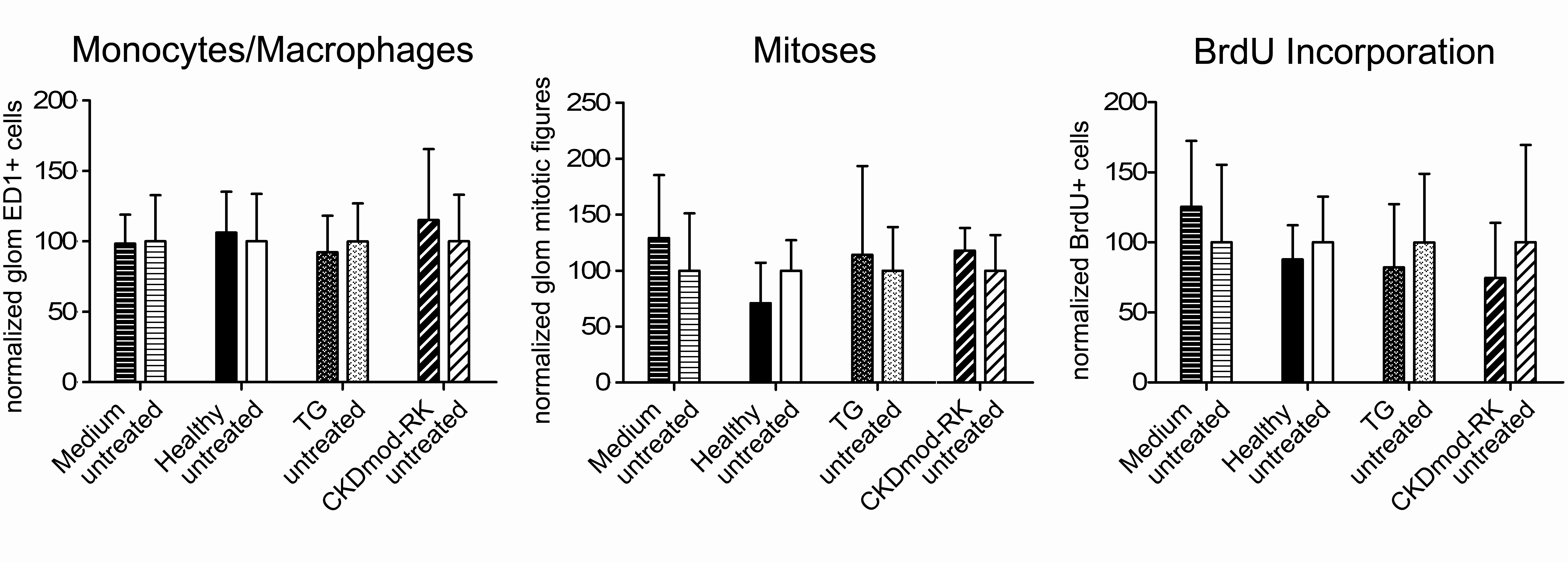


Comparison of rats that had anti-Thy1.1-nephritis and received H-MSCs, TG-MSCs, CKDmod-RK-MSCs or control medium into the left renal artery on day 2 after disease induction.

* p < 0.05. All data: mean ± SD.
