# Supplementary material for: Mesenchymal Stem Cells from Rats with Chronic Kidney Disease Exhibit Premature Senescence and Loss of Regenerative Potential
Source: PLoS One. 2014 Mar 25;9(3):e92115. doi: 10.1371/journal.pone.0092115 (PMC3965415; doi:10.1371/journal.pone.0092115)
Supplement: Figure S8 — Analysis of renal histology on day 6 of anti-Thy1.1-nephritis treated with CKDsev-AD-MSCs. (DOC) [file pone.0092115.s008.doc]

**Supplementary Figure S11:**

**Analysis of renal histology on day 6 of anti-Thy1.1-nephritis**

**treated with CKDsev-AD-MSCs**


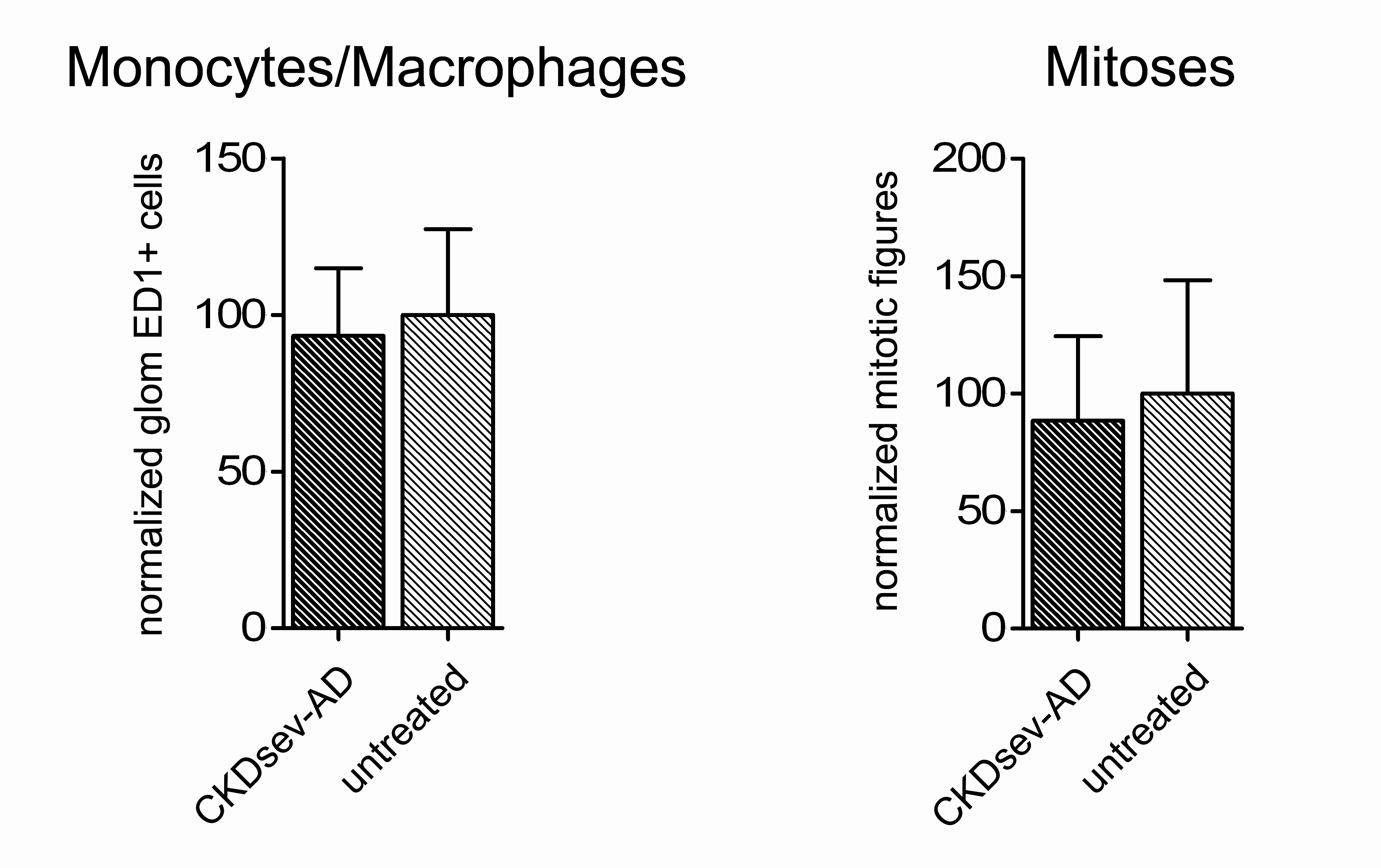


Comparison of left and right kidneys of rats that had anti-Thy1.1-nephritis and received CKDsev-AD-MSCs into the left renal artery on day 2 after disease induction.

* p < 0.05. All data: mean ± SD.
