# Supplementary material for: Mesenchymal Stem Cells from Rats with Chronic Kidney Disease Exhibit Premature Senescence and Loss of Regenerative Potential
Source: PLoS One. 2014 Mar 25;9(3):e92115. doi: 10.1371/journal.pone.0092115 (PMC3965415; doi:10.1371/journal.pone.0092115)
Supplement: File S1 — Western Blot for intracellular accumulation of actin filaments in MSC (method in detail). (DOC) [file pone.0092115.s009.doc]

Supplementary File S1:

Western Blot for intracellular accumulation of actin filaments in MSC

Method in detail

Accumulation of actin fibers within cells is regarded a marker of cellular senescence. Total cellular protein lysates were prepared by homogenizing subconfluent P2 MSC in lysis buffer (containing 50mM Hepes, 150 mM NaCl, 1,5 mM MgCl_2_, 1 mM EGTA, 10% Glycerin, 1% Triton X-100, 1% protease-/phosphatase inhibitor (Halt TM Protease and Phosphatase Inhibitor, Single-Use Cocktail, EDTA-free, Thermo Scientific, Rockford, IL) at 4°C. Incubation for 5 min preceeded ultrasound treatment (3x10s). After centrifugation at 14000 rpm, the concentrations of soluable proteins in the resulting supernatants were determined using the BCA protein assay (Protein quantication kit, Uptima, Interchim, Montluçon, Cedex, France).

Four µg of protein were electrophoresed under reducing conditions on a NuPAGE 4-12% Bis-Tris gel (Invitrogen, Carlsbad, USA) in NuPAGE MES SDS running buffer (Invitrogen, Carlsbad, USA) and then blotted onto nitrocellulose membranes. MagicMark XP was used as protein standard (Invitrogen, Carlsbad, USA). The blots were blocked with 2% BSA (Sigma) in TTBS (150mM NaCl, 10mM Tris, pH 8.0, 0.05% Tween 20) for 1h at room temperature and then incubated with an actin mouse monoclonal antibody (sc-8432, Santa Cruz Biotechnology, Santa Cruz, USA) diluted in TTBS overnight at 4°C. After several washes with TTBS, the blots were incubated with a horseradish peroxidase-conjugated horse-anti-mouse antibody (Vector Laboratories, Burlingame, CA, USA) for 1 h. The blots were visualized with the enhanced chemiluminescence reagent ECL (Pierce, Thermo Scientific, Rockford, USA). To ensure equal protein loading, membranes were stripped, washed and blocked with 2% BSA again followed by incubation with a mouse monoclonal antibody for Glyceraldehyde-3-phosphate dehydrogenase (GAPDH) (sc-32233, Santa Cruz Biotechnology, Santa Cruz, USA) visualization of the result as described above. Stripping was necessary as the proteins of interest (actin and GAPDH) have a predicted size of 42 and 36-40 kD, respectively. Band intensities were quantified by Scion Image software (Scion Corporation, USA), and after normalization against values determined for GAPDH, the actin content in healthy control MSC was set as 1 and relative band intensities were calculated.
