# Supplementary material for: Mesenchymal Stem Cells from Rats with Chronic Kidney Disease Exhibit Premature Senescence and Loss of Regenerative Potential
Source: PLoS One. 2014 Mar 25;9(3):e92115. doi: 10.1371/journal.pone.0092115 (PMC3965415; doi:10.1371/journal.pone.0092115)
Supplement: Table S1 — Primer for RT-qPCR. (DOC) [file pone.0092115.s011.doc]

**Supplementary Table S2:**

**Primer for RT-qPCR**

| rat gene | forward primer | reverse primer | taqman probe |
| --- | --- | --- | --- |
| Gapdh | 5’-ACAAGATGGTGAAGGTCGGTG-3’ | 5’-AGAAGGCAGCCCTGGTAACC-3’ | 5’-CGGATTTGGCCGTAT CGGACGC-3’ |
| Osteopontin | 5’-CGGCCTTGCCTCCTGTCT-3’ | 5’-AATGCGCCTTCTCCTCTGAG-3’ | --- |
| Cbfa1 | 5’-TGCAACAAGACCCTGCCC-3’ | 5’-ACGGTCCCATCGGGTACC-3’ | --- |
| Adiponectin | 5’-GGGATTACTGCAACCGAAGG-3’ | 5’-CCATCCAACCTGCACAAGTTT-3’ | --- |
| Lipoprotein lipase | 5’-GTACAGTCTTGGAGCCCATGC-3’ | 5’-GCCAGTAATTCTATTGACCTTCTTGTT-3’ | --- |
| PPAR-γ | 5’-CATACATAAAGTCCTTCCCGCTG-3’ | 5’-TTGTCTGTTGTCTTTCCTGTCAAGA-3’ | --- |
| Bglap (osteocalcin) | 5’-TGAGCTCAACCCCAATTGTG-3’ | 5’-GCGCTTGTAGGCGTCCTG-3’ | --- |
| PDGF-A | 5’-TTCTTGATCTGGCCCCCAT-3’ | 5’-TTGACGCTGCTGGTGTTACAG-3’ | 5’-CAGTGCAGCGCTTCACCT CCACA-3’ |
| PDGF-B | 5’-GCAAGACGCGTACAGAGGTG-3’ | 5’-GAAGTTGGCATTGGTGCGA-3’ | 5’-TCCAGATCTCGCGGAACC TCATCG-3’ |
| PDGF-C | 5’-CAGCAAGTTGCAGCTCTCCA-3’ | 5’-GACAACTCTCTCATGCCGGG-3’ | 5’-CGACAAGGAGCAGAA CGGAGTGCAA-3’ |
| PDGF-D | 5’-ATCGGGACACTTTTGCGACT-3’ | 5’-GTGCCTGTCACCCGAATGTT-3’ | 5’-TTGCGCAATGCCAACCTC AGGAG-3’ |
| PDGFRα | 5`-GCCACGAAAGAGGTCAAGGA-3` | 5`- GCCTGATCTGGACGAAGCC-3` | 5`-TGAAGACAGTCACCATTTCTG TTCACGAGAA-3’ |
| PDGFRβ | 5’-AATGACCACGGCGATGAGA-3’ | 5’-TCTTCCAGTGTTTCCAGCAGC-3’ | 5’-CATCAACGTTACTGTGATCGA AAATGGCTATG-3’ |
| Collagen I α I | 5’-GAAGGCAACAGTCGATTCACC-3’ | 5’- GACTGTCTTGCCCCAAGTTCC-3’ | --- |
| Collagen III α I | 5’- GAAGGCAACAGTCGATTCACC-3’ | 5’- TCCCGAGTCGCAGACACATA-3’ | 5’-TGACATGGTTCTGGCTTC CAGACATCTCTAG-3’ |
| Gas7 | 5`-TTGGCAGAGAACTTGAGGTGAA-3` | 5`- GAGGCATGGGCCCAAGTA-3´ |  |
