# Supplementary material for: Mesenchymal Stem Cells from Rats with Chronic Kidney Disease Exhibit Premature Senescence and Loss of Regenerative Potential
Source: PLoS One. 2014 Mar 25;9(3):e92115. doi: 10.1371/journal.pone.0092115 (PMC3965415; doi:10.1371/journal.pone.0092115)
Supplement: Table S2 — mRNA expression of osteogenic markers in MSCs. (DOC) [file pone.0092115.s012.doc]

**Supplementary Table S3**

**mRNA expression of osteogenic markers in MSCs**

**All values are normalized to H-MSCs. Data: mean ± SD.**

|  | **H-MSC** | **CKDmod-RK-MSC** | **CKDsev-AD-MSC** | **old donor MSC** | **CKDsev-AD-MSC** |
| --- | --- | --- | --- | --- | --- |
|  | (n = 7) | (n = 6) | (n = 5) | (n = 6) | (n = 6) |
| Cbfα1 (core-binding factor subunit α1) | 1 ± 0.34 | 2.09 ± 0.99 | 2.47 ± 1.36 | 1.43 ± 0.93 | 0.70 ± 0.28 |
| Osteopontin | 1 ± 0.75 | 2.47 ± 1.69 | 1.82 ± 2.10 | 0.57 ± 0.52 | 1.27 ± 0.90 |
| Osteocalcin | 1 ± 0.54 | 7.33 ± 3.75 * | 5.34 ± 6.68 | 1.24 ± 1.09 | 0.72 ± 0.57 |

* p < 0.05 compared to H-MSC and old donor-MSC.
